# BAG-1 haplo-insufficiency impairs lung tumorigenesis

**DOI:** 10.1186/1471-2407-4-85

**Published:** 2004-11-24

**Authors:** Rudolf Götz, Boris W Kramer, Guadalupe Camarero, Ulf R Rapp

**Affiliations:** 1 Institut für Medizinische Strahlenkunde und Zellforschung (MSZ), Universität Würzburg, Versbacher Straße 5, D-97078 Würzburg, Germany; 2 Universitäts-Kinderklinik Würzburg, Josef-Schneider-Str. 2, D-97080 Würzburg, Germany

## Abstract

**Background:**

BAG-1 is a multifunctional co-chaperone of heat shock proteins (Hsc70/Hsp70) that is expressed in most cells. It interacts with Bcl-2 and Raf indicating that it might connect protein folding with other signaling pathways. Evidence that BAG-1 expression is frequently altered in human cancers, in particular in breast cancer, relative to normal cells has been put forward but the notion that overexpression of BAG-1 contributes to poor prognosis in tumorigenesis remains controversial.

**Methods:**

We have evaluated the effect of BAG-1 heterozygosity in mice in a model of non-small-cell lung tumorigenesis with histological and molecular methods. We have generated mice heterozygous for BAG-1, carrying a BAG-1 null allele, that in addition express oncogenic, constitutively active C-Raf kinase (SP-C C-Raf BxB) in type II pneumocytes. SP-C C-Raf BxB mice develop multifocal adenomas early in adulthood.

**Results:**

We show that BAG-1 heterozygosity in mice impairs C-Raf oncogene-induced lung adenoma growth. Lung tumor initiation was reduced by half in BAG-1 heterozygous SP-C C-Raf BxB mice compared to their littermates. Tumor area was reduced by 75% in 4 month lungs of BAG-1 haploinsufficient mice compared to mice with two BAG-1 copies. Whereas BAG-1 heterozygosity did not affect the rate of cell proliferation or signaling through the mitogenic cascade in adenoma cells, it increased the rate of apoptosis.

**Conclusion:**

Reduced BAG-1 expression specifically targets tumor cells to apoptosis and impairs tumorigenesis. Our data implicate BAG-1 as a key player in oncogenic transformation by Raf and identify it as a potential molecular target for cancer treatment.

## Background

BAG-1 is a multifunctional protein that is expressed in most cells. Originally identified as a Bcl-2 binding protein [1], other interaction partners of BAG-1 were described, including the serine threonine kinase C-Raf [2]. The C-terminal "BAG domain" of BAG-1 mediates the interaction with the Hsc70 and Hsp70 heat shock proteins [3], molecular chaperones that bind proteins in non-native states assisting them to reach a functional active conformation [4]. BAG-1 acts as a nucleotide exchange factor in this activation cycle [3]. The above findings indicated that BAG-1 might connect protein folding with other signaling pathways. Signaling networks promoting cell growth and proliferation are frequently deregulated in cancer [5]. The classical mitogenic cascade transmits stimuli from growth factor receptors via Ras, Raf, MEK and ERK to the cell nucleus [6]. C-Raf, like A- and B-Raf kinases also act at the outer membrane of mitochondria to augment cell survival [7,8]. Previously we had observed the stimulation of C-Raf kinase activity by BAG-1 in vitro [2]. Ras and B-Raf mutations have been found in various human cancers [9,10]. Evidence that BAG-1 expression is frequently altered in human cancers, in particular in breast cancer, relative to normal cells has been put forward but the notion that overexpression of BAG-1 contributes to poor prognosis in tumorigenesis remains controversial [11].

## Methods

### Animals

Mice used in these studies were generated and maintained according to protocols approved by the animal care and use committee at University of Würzburg. To inactivate the BAG-1 gene, we constructed a vector where exons 1 and 2 are replaced with a neomycin resistance gene. A phage clone with a 15-kb genomic insert from mouse strain 129/Sv spanning all seven exons of BAG-1 was identified and characterised using standard methods. The targeting construct contained 1,1-kb from the BAG-1 locus upstream of the neomycin resistance gene of plasmid pPNT [12] and 6-kb downstream. The upstream arm of 1,1 kb is located 5' to the start codon in the first exon of BAG-1 and the 3' arm of 6 kb is located downstream of exon 2. The mutation was introduced into embryonic stem cells by homologous recombination. Positive clones were identified by Southern blot analysis. Germline transmitting chimeras were obtained and bred to C57BL/6 mice. Further details will be described elsewhere. Heterozygous BAG-1 mice were genotyped by a PCR assay. The targeted BAG-1 allele was detected with primers P1 (5'-GAG TCT CCC GAT CCC TTT TCC), located upstream of exon 1, and P2 (5'-GAT TCG CAG CGC ATC GCC TT), located in the neomycin resistance gene, yielding a product of 600 base pairs. BAG-1 heterozygous mice were backcrossed at least three times onto C57BL/6 background before crossing with SP-C C-Raf BxB mice. Lung tumour mice expressing oncogenic C-Raf BxB were backcrossed at least six times onto C57BL/6 background.

### Western blot

For the analysis of BAG-1 expression, lung lysates of the indicated genotypes were separated on 12,5% polyacrylamide-SDS (sodium dodecyl sulphate) gel, transferred to nitrocellulose Protran BA83 membrane (Schleicher&Schüll) and probed with rabbit anti-BAG-1 (FL-274) antibody (1:250, Santa Cruz Biotechnology). Amounts of protein were determined by Bradford protein assay to ensure equal protein loading for the analysis. Blots were developed using the appropriate horseradish peroxidase coupled secondary antibody and the ECL system (Amersham Pharmacia Biotech). Subsequently, the membrane was stripped and reprobed with rabbit antibody to glyceraldehyde 3 phosphate dehydrogense (1:2000, ab9485, Abcam Ltd.).

### Histopathology and immunohistochemistry

Animals were sacrificed and lungs were fixed under 25 cm water pressure with 4% paraformaldehyde and embedded in paraffin. 5 μm sections were stained with hematoxylin and eosin and analysed. Pictures were taken using a Leica DMLA microscope and a Hitachi HV-C20A colour camera. Immunohistochemical staining to detect activated caspase-3, phospho-ERK (extracellular signal-regulated kinase), PCNA (proliferating cell nuclear antigen) have been described elsewhere [13]. Apoptotic, PCNA and p-ERK indices were determined by evaluating randomly chosen adenomas or fields of normal lung in 3–4 sections and determining the percentage of positive cells per 2000 cells at ×400.

## Results and discussion

### BAG-1 heterozygosity impairs C-Raf driven tumorigenesis

In order to assess the functional role of BAG-1 on tumorigenesis, we have generated a null allele of BAG-1. To inactivate the BAG-1 gene, exons 1 and 2 were replaced with a neomycin resistance gene. This strategy was chosen to disrupt the expression of all known isoforms of BAG-1 which are generated by alternate translation initiation of a single mRNA; the start codons are present in exons 1 and 2. Western blot analysis of liver protein extracts of BAG-1 deficient embryos showed the complete loss of all BAG-1 protein isoforms. Embryos homozygous for this allele died at midgestation at around E13,5, but the heterozygous animals (BAG-1^+/-^) are normal. A comprehensive description of the BAG-1^-/- ^phenotype is subject of another manuscript.

**Figure 1:**
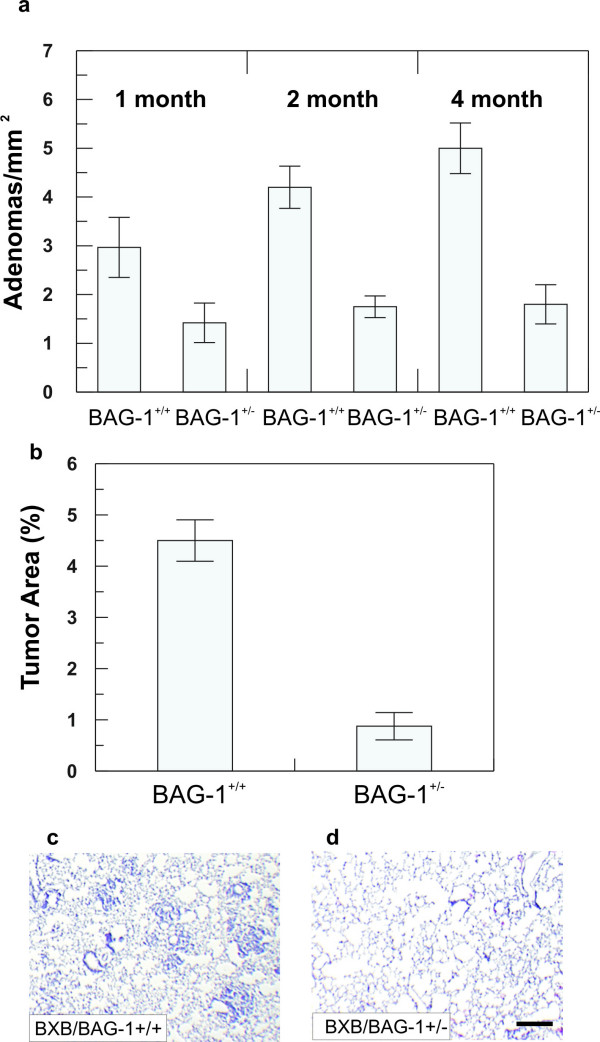
**BAG-1 haplo-insufficiency delays C-Raf driven adenoma growth. **(**a**) Adenoma initiation in SP-C C-Raf BxB mice (BAG-1^+/+^) and their Bag-1 haplo-insufficient littermates (BAG-1^+/-^) at 1, 2 and 4 months of age. Adenoma foci values represent mean ± s.e. from at least 4 mice of each genotype analyzed in a blinded fashion by two independent readers. (**b**) Adenoma area in the lungs of 4 months old mice. Each value represents mean ± s.e. from at least 4 mice of each genotype analyzed in a blinded fashion. (**c-d**) Examples of hematoxylin-eosin stained sections of lungs from SP-C C-Raf BxB transgenic mice wildtype for BAG-1 in comparison to a BAG-1 heterozygous littermate. Scale bar, 200 μm.

Previously, we had generated a lung cancer mouse model by targeting constitutively active C-Raf kinase (SP-C C-Raf BxB) to the lung [14]. These mice develop multifocal adenomas early in adulthood. Based on the observation, that BAG-1 can activate C-Raf [2], we asked whether heterozygosity for BAG-1 would affect C-Raf BxB driven adenoma growth. We observed that lung tumour initiation was reduced by half in 1, 2 and 4 months old BAG-1^+/- ^mice transgenic for SP-C C-Raf BxB compared to their BAG-1^+/+ ^littermates. Tumour area was reduced by 75% in 4 month lungs of BAG-1 haploinsufficient mice compared to mice with two BAG-1 copies, see Figure 1. The histological picture emphasises the difference in adenoma formation between a representative SP-C C-Raf BxB/BAG-1^+/+ ^and SP-C C-Raf BxB/BAG-1^+/- ^lung. The difference in the staining intensity of the two lung sections derives mainly from the observation that the adenoma cells have a tendency to bind more intensively hematoxylin and eosin compared to normal lung cells. Thus, reduction of the BAG-1 gene dosage impairs the oncogenic activity of C-Raf in vivo.

### Reduced BAG-1 expression in BAG-1 heterozygous lungs

**Figure 2:**
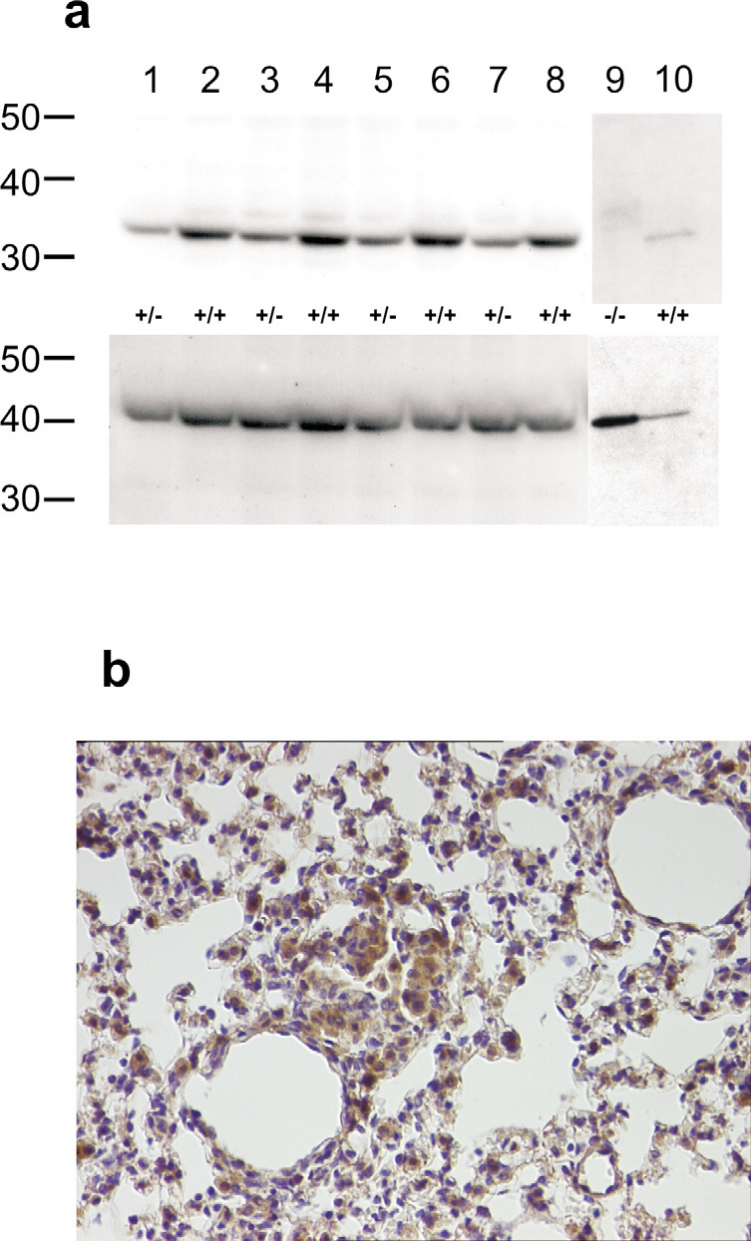
**BAG-1 expression in the lung of SP-C C-Raf BxB transgenic mice **(**a**) Lanes 1–8 show immunoblotting data for expression of BAG-1 in the lungs of 8 month old SP-C C-Raf BxB transgenic mice heterozygous (+/-) or homozygous (+/+) for BAG-1 as indicated below the lanes. Lane 9 shows absence of BAG-1 expression in a BAG-1 null (-/-) embryonic day 12,5 liver extract; lane 10 control liver. The markers along the left indicate relative molecular mass. The same blots were subsequently reacted with an antibody against GAPDH to demonstrate protein equal loading and are shown below. (**b**) BAG-1 immunostaining in SP-C C-Raf BxB transgenic mouse lung cancer tissue.

Quantitative immunoblots demonstrated that the specific BAG-1 protein concentration in the lungs of BAG-1^+/- ^mice was half the amount of BAG-1^+/+ ^littermates, see Figure 2a. Moreover, immunohistochemical staining showed that BAG-1 was expressed in adenoma cells, see Figure 2b. There was no obvious difference in the BAG-1 immunohistochemistry of SP-C C-RafBxB/BAG-1^+/+ ^and SP-C C-RafBxB/BAG-1^+/- ^lungs.

### Tumour cells of BAG-1 heterozygous mice show increased apoptosis

**Figure 3:**
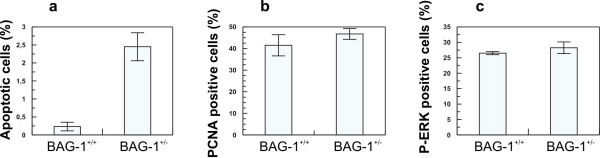
**Increased apoptosis but no change in PCNA and p-ERK in tumor cells of SP-C C-RafBxB transgenic BAG-1 heterozygous mice **(**a-c**) Quantification of immunohistochemical staining for apoptosis using an antibody that detects activated caspase-3 (**a**), PCNA (**b**) and phosphorylated ERK (p-ERK, **c**) of adenoma cells from 1-month-old SP-C C-Raf BxB transgenic mice of the indicated BAG-1 genotype. Each value represents mean ± s.e. from at least 4 mice of each genotype analyzed in three different experiments.

Concerning the molecular mechanism how a reduction of the BAG-1 protein expression in the heterozygous mice would impair tumorigenesis, we determined the fraction of apoptotic cells. Staining for activated caspase-3 revealed indistinguishable apoptosis in healthy regions of the lung of 1 month old SP-C C-Raf BxB mice with either one or two BAG-1 alleles, in line with the unaltered, normal lung structure of BAG-1^+/- ^mice. In the adenomas, however, we observed a significant increase of apoptotic cells in BAG-1^+/- ^SP-C C-Raf BxB mice compared with their SP-C C-Raf BxB/BAG-1^+/+ ^littermates, see Figure 3a. This mechanism of action of BAG-1 on the regulation of cell survival is compatible with the phenotype of embryonic day 12,5 BAG-1 null embryos. Immunohistochemical staining for activated caspase-3 and trypan blue staining of dissociated cells showed hypocellularity and elevated levels of apoptosis in the livers of BAG-1^-/- ^embryos (unpublished observations).

### Proliferation and p-ERK signalling are unaffected in BAG-1 heterozygous mice

To exclude the alternative mechanism that the decreased level of BAG-1 expression in heterozygous animals would cause reduced cell proliferation in the adenomas, we performed proliferating cellular antigen (PCNA) staining. No significant differences were observed in the fraction of proliferating adenoma cells between SP-C C-Raf BxB animals heterozygous or wild type for BAG-1, see Figure 3b. Also, the percentages of adenoma cells positive for Ki-67, another proliferation marker and Bmi-1, a chromatin-associated protein expressed in stem cells, were not affected by the BAG-1 heterozygosity (not shown). Furthermore, staining of lung sections for phosphorylated ERK revealed no quantitative differences in the adenomas of SP-C C-Raf BxB animals heterozygous or wild type for BAG-1, see Figure 3c. Thus, signalling through the mitogenic cascade was not affected by the BAG-1 heterozygosity in the adenoma cells.

## Conclusions

Tumours often are highly dependent on signalling pathways promoting cell growth or survival and may become hypersensitive to downregulation of key components within these signalling cascades. This study identifies BAG-1 as a protein specifically required at wild type expression levels for the survival of tumour cells and reveals it as potential anticancer target. Since many key components of survival pathways are regulated by interaction with (co-)chaperones [15], our finding is not without precedent but novel insofar as we have uncovered that reduced BAG-1 expression specifically targets tumour cells to apoptosis and impairs tumorigenesis. Whether this effect on adenoma cell survival requires that BAG-1 interacts with C-Raf or Hsc70/Hsp70 or with both partners requires additional studies. Questions concerning specific roles of the different BAG-1 isoforms were not addressed with this BAG-1 deficient mouse as both isoforms of BAG-1, p50 and p32 are absent in protein extracts of knock-out embryos. Another setting where BAG-1 has a physiological role is the heart, where up-regulation of BAG-1 after ischemia rescues cells from apoptosis [16].

**Figure 4:**
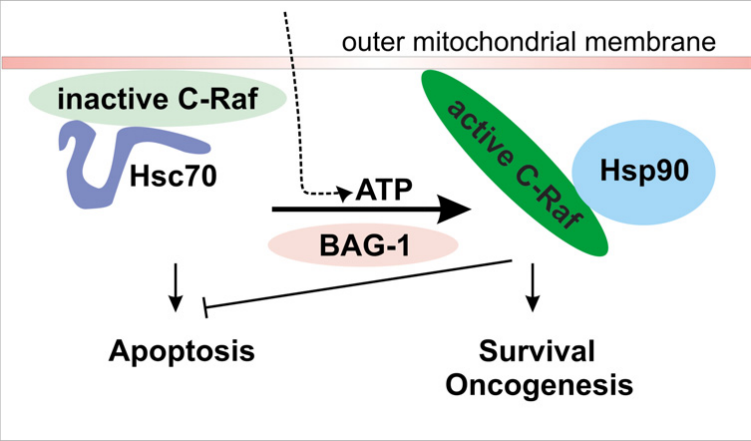
**Model for cooperative action of BAG-1 and C-Raf in tumorigenesis **A possible model combining the findings of this report and other data is shown. It indicates that BAG-1 functions as an activator of C-Raf at the outer mitochondrial membrane where enzymatically activated C-Raf finds apoptosis-related targets (for details see text).

A possible model combining the findings of this report and other data indicates that BAG-1 functions as an activator of C-Raf at the outer mitochondrial membrane where enzymatically activated C-Raf finds apoptosis-related targets such as BAD [17], see Figure 4. We can purify overexpressed C-Raf either in an enzymatically inactive form in a complex with Hsp70 or in an enzymatically active form in a complex with Hsp90/50 (unpublished observations), and BAG-1 is proposed to regulate this activation with ATP generated in the mitochondria. Experiments dealing with this questions are currently ongoing.

Therefore, the therapeutic efficacy of a standard chemotherapeutic agent [13] should be increased dramatically by co-application with a BAG-1 inhibitor, since it would target the adaptability of cancer cells to environmental stress and overcome their genetic plasticity. One way to reduce BAG-1 expression is through use of RNA interference-based gene silencing, in particular as BAG-1 overexpression has been observed in human tumours [11]. Drugs that bind to the ATP binding site of Hsc70/Hsp70 might also be expected to be effective as they would inhibit the interaction of BAG-1 with the ATPase domain of heat shock proteins. Such new specific BAG-1 inhibitors may be identified, aided by the known three-dimensional structure of the BAG domain [18,19].

## Competing interests

The author(s) declare that they have no competing interests.

## Authors' contributions

RG caried out the molecular and histological studies and participated in the design and co-ordination of the study. BWK carried out the histological and immunohisto-chemical studies. GC participated in the histological and immunohistochemical experiments. URR participated in the design and co-ordination of the study. All authors read and approved the final manuscript.

## Pre-publication history

The pre-publication history for this paper can be accessed here:



## References

[B1] Takayama S, Sato T, Krajewski S, Kochel K, Irie S, Millan JA, Reed JC (1995). Cloning and functional analysis of BAG-1:  A novel Bcl-2- binding protein with anti-cell death activity. Cell.

[B2] Wang HG, Takayama S, Rapp UR, Reed JC (1996). Bcl-2 interacting protein, BAG-1, binds to and activates the kinase Raf-1. Proc Natl Acad Sci USA.

[B3] Townsend PA, Cutress RI, Sharp A, Brimmell M, Packham G (2003). BAG-1: a multifunctional regulator of cell growth and survival. Biochim Biophys Acta.

[B4] Mayer MP, Brehmer D, Gassler CS, Bukau B (2001). Hsp70 chaperone machines. Adv Protein Chem.

[B5] Weinstein IB (2002). Cancer. Addiction to oncogenes--the Achilles heal of cancer. Science.

[B6] Avruch J, Khokhlatchev A, Kyriakis JM, Luo Z, Tzivion G, Vavvas D, Zhang XF (2001). Ras activation of the Raf kinase: tyrosine kinase recruitment of the MAP kinase cascade. Recent Prog Horm Res.

[B7] Wang HG, Rapp UR, Reed JC (1996). Bcl-2 targets the protein kinase Raf-1 to mitochondria. Cell.

[B8] Alavi A, Hood JD, Frausto R, Stupack DG, Cheresh DA (2003). Role of Raf in vascular protection from distinct apoptotic stimuli. Science.

[B9] Jr JT, McCubrey JA (2002). Targeting the Raf kinase cascade in cancer therapy - novel molecular targets and therapeutic strategies. Expert Opin Ther Targets.

[B10] Davies H, Bignell GR, Cox C, Stephens P, Edkins S, Clegg S, Teague J, Woffendin H, Garnett MJ, Bottomley W, Davis N, Dicks E, Ewing R, Floyd Y, Gray K, Hall S, Hawes R, Hughes J, Kosmidou V, Menzies A, Mould C, Parker A, Stevens C, Watt S, Hooper S, Wilson R, Jayatilake H, Gusterson BA, Cooper C, Shipley J, Hargrave D, Pritchard-Jones K, Maitland N, Chenevix-Trench G, Riggins GJ, Bigner DD, Palmieri G, Cossu A, Flanagan A, Nicholson A, Ho JW, Leung SY, Yuen ST, Weber BL, Seigler HF, Darrow TL, Paterson H, Marais R, Marshall CJ, Wooster R, Stratton MR, Futreal PA (2002). Mutations of the BRAF gene in human cancer. Nature (London).

[B11] Cutress RI, Townsend PA, Brimmell M, Bateman AC, Hague A, Packham G (2002). BAG-1 expression and function in human cancer. Br J Cancer.

[B12] Tybulewicz VLJ, Crawford CE, Jackson PK, Bronson RT, Mulligan RC (1991). Neonatal lethality and lymphopenia in mice with a homozygous disruption of the c-abl proto-oncogene. Cell.

[B13] Kramer BW, Götz R, Rapp UR (2004). Use of mitogenic cascade blockers for treatment of C-Raf induced lung adenoma in vivo: CI-1040 strongly reduces growth and improves lung structure. BMC Cancer.

[B14] Kerkhoff E, Fedorov LM, Siefken R, Walter AO, Papadopoulus T, Rapp UR (2000). Lung-targeted expression of the c-Raf-1 kinase in transgenic mice exposes a novel oncogenic character of the wild-type protein. Cell Growth Diff.

[B15] Takayama S, Reed JC, Homma S (2003). Heat-shock proteins as regulators of apoptosis. Oncogene.

[B16] Townsend PA, Cutress RI, Carroll CJ, Lawrence KM, Scarabelli TM, Packham G, Stephanou A, Latchman DS (2004). BAG-1 proteins protect cardiac myocytes from simulated ischemia/reperfusion-induced apoptosis via an alternate mechanism of cell survival independent of the proteasome. J Biol Chem.

[B17] Rapp UR, Rennefahrt U, Troppmair J (2004). Bcl-2 proteins: master switches at the intersection of death signaling and the survival control by Raf kinases. Biochim Biophys Acta.

[B18] Briknarova K, Takayama S, Brive L, Havert ML, Knee DA, Velasco J, Homma S, Cabezas E, Stuart J, Hoyt DW, Satterthwait AC, Llinas M, Reed JC, Ely KR (2001). Structural analysis of BAG1 cochaperone and its interactions with Hsc70 heat shock protein. Nat Struct Biol.

[B19] Sondermann H, Scheufler C, Schneider C, Höhfeld J, Hartl FU, Moarefi I (2001). Structure of a Bag/Hsc70 complex: Convergent functional evolution of Hsp70 nucleotide exchange factors. Science.

